# Antimicrobial Resistance in Invasive Non-typhoid *Salmonella* from the Democratic Republic of the Congo: Emergence of Decreased Fluoroquinolone Susceptibility and Extended-spectrum Beta Lactamases

**DOI:** 10.1371/journal.pntd.0002103

**Published:** 2013-03-14

**Authors:** Octavie Lunguya, Veerle Lejon, Marie-France Phoba, Sophie Bertrand, Raymond Vanhoof, Youri Glupczynski, Jan Verhaegen, Jean-Jacques Muyembe-Tamfum, Jan Jacobs

**Affiliations:** 1 National Institute for Biomedical Research, Kinshasa, Democratic Republic of the Congo; 2 University Hospital of Kinshasa, Kinshasa, Democratic Republic of the Congo; 3 Department of Clinical Sciences, Institute of Tropical Medicine, Antwerp, Belgium; 4 Institut de Recherche pour le Développement, UMR 177 IRD-CIRAD INTERTRYP, Campus International de Baillarguet, Montpellier, France; 5 Institute of Public Health, Brussels, Belgium; 6 National Reference Laboratory for Monitoring of Antimicrobial Resistance in Gram-negative Bacteria, Cliniques Universitaires UCL de Mont-Godinne, Yvoir, Belgium; 7 University Hospital Leuven, Leuven, Belgium; Oxford University Clinical Research Unit, Viet Nam

## Abstract

**Background:**

Co-resistance against the first-line antibiotics ampicillin, chloramphenicol and trimethoprim/sulphamethoxazole or multidrug resistance (MDR) is common in non typhoid *Salmonella* (NTS). Use of alternative antibiotics, such as fluoroquinolones or third generation cephalosporins is threatened by increasing resistance, but remains poorly documented in Central-Africa.

**Methodology/Principal findings:**

As part of a microbiological surveillance study in DR Congo, blood cultures were collected between 2007 and 2011. Isolated NTS were assessed for serotype and antimicrobial resistance including decreased ciprofloxacin susceptibility and extended-spectrum beta-lactamase (ESBL) production. In total, 233 NTS isolates (representing 23.6% of clinically significant organisms) were collected, mainly consisting of *Salmonella* Typhimurium (79%) and *Salmonella* Enteritidis (18%). The majority of NTS were isolated in the rainy season, and recovered from children ≤2 years old. MDR, decreased ciprofloxacin susceptibility, azithromycin and cefotaxime resistance were 80.7%, 4.3%, 3.0% and 2.1% respectively. ESBL production was noted in three (1.3%) isolates. Decreased ciprofloxacin susceptibility was associated with mutations in codon 87 of the *gyrA* gene, while ESBLs all belonged to the SHV-2a type.

**Conclusions/Significance:**

Presence of almost full MDR among NTS isolates from blood cultures in Central Africa was confirmed. Resistance to fluoroquinolones, azithromycin and third generation cephalosporins is still low, but emerging. Increased microbiological surveillance in DR Congo is crucial for adapted antibiotic therapy and the development of treatment guidelines.

## Author Summary

Invasive non typhoid *Salmonella spp*. (NTS) are an important cause of bloodstream infection in sub-Saharan Africa and associated with a high mortality. Levels of multidrug resistance have become alarmingly high. Treatment therefore increasingly relies on the oral fluoroquinolones such as ciprofloxacin, with third generation cephalosporins such as cefotaxime as alternatives for parenteral treatment. Azithromycin represents another alternative antimicrobial drug. Worldwide, increased use of these drugs is associated with spread of resistance as well, a phenomenon poorly documented in Central-Africa. In the present study, 233 NTS isolates were collected from blood cultures sampled between 2007 and 2011 in DR Congo, mainly from children ≤2 years of age. Most isolates were recovered during the rainy season. Widespread multidrug resistance was confirmed as well as decreased susceptibility to ciprofloxacin, resistance to azithromycin and resistance to third generation cephalosporins. Our findings demonstrate emergence of antibiotic resistance among NTS in DR Congo and underline the need for increased microbiological surveillance, being a prerequisite for rational antibiotic therapy and the development of standard treatment guidelines.

## Introduction

Non typhoid *Salmonella* (NTS) are among the leading causes of bacterial bloodstream infections in sub-Saharan Africa [1], [2]. NTS bacteremia mainly affects immune compromised hosts and young children, in whom they are associated with high mortality rates up to 27% [3]. Usually, most cases of invasive non typhoid salmonellosis are due to either *Salmonella enterica* subsp. *enterica* serotype Typhimurium (further referred to as *Salmonella* Typhimurium), followed by *Salmonella* Enteritidis [2]. Resistance of NTS to the first line antibiotics ampicillin, chloramphenicol and trimethoprim/sulphamethoxazole (TMP-SMX) is usually high [4], [5], also in the Democratic Republic of the Congo (DR Congo) [6]–[9]. Treatment of NTS therefore increasingly relies on fluoroquinolones or third generation cephalosporins but these treatment options are threatened by decreased susceptibility to fluoroquinolones (referred to as decreased ciprofloxacin susceptibility) and extended-spectrum beta-lactamases (ESBLs) respectively. In Central Africa, decreased ciprofloxacin susceptibility in NTS is poorly documented [8], [10], [11] and NTS resistance to third generation cephalosporins in NTS has not yet been described [7], [10], in contrast to other regions in tropical Africa [12]–[15].

The present study describes the antimicrobial resistance profile of invasive NTS isolates recovered from bloodstream infections during a microbiological surveillance study in DR Congo over the years 2007–2011. The molecular mechanisms of decreased ciprofloxacin susceptibility and ESBLs were characterized and the genetic relationships of *Salmonella* Enteritidis and *Salmonella* Typhimurium isolates were assessed.

## Materials and Methods

### Ethics statement

Ethical approval was granted by the Ethical Committee of the University of Antwerp, Belgium and from the Ministry of Health in DR Congo. The present study complies with the World Health Organization and international guidelines (European Society of Clinical Microbiology and Infectious Diseases Study Group for Antimicrobial Resistance Surveillance and Clinical Laboratory Standards Institute) on antibiotic surveillance for which no recommendation for an informed consent has been issued. The diagnostic procedure – blood cultures – is part of the standard diagnostic work-up of patients with a suspicion of bacteremia. Clinical information -as presented- and information about use of antibiotics was the standard information present on the laboratory request form. Data have been reviewed and analysed anonymously.

### Study setting

**Figure 1:**
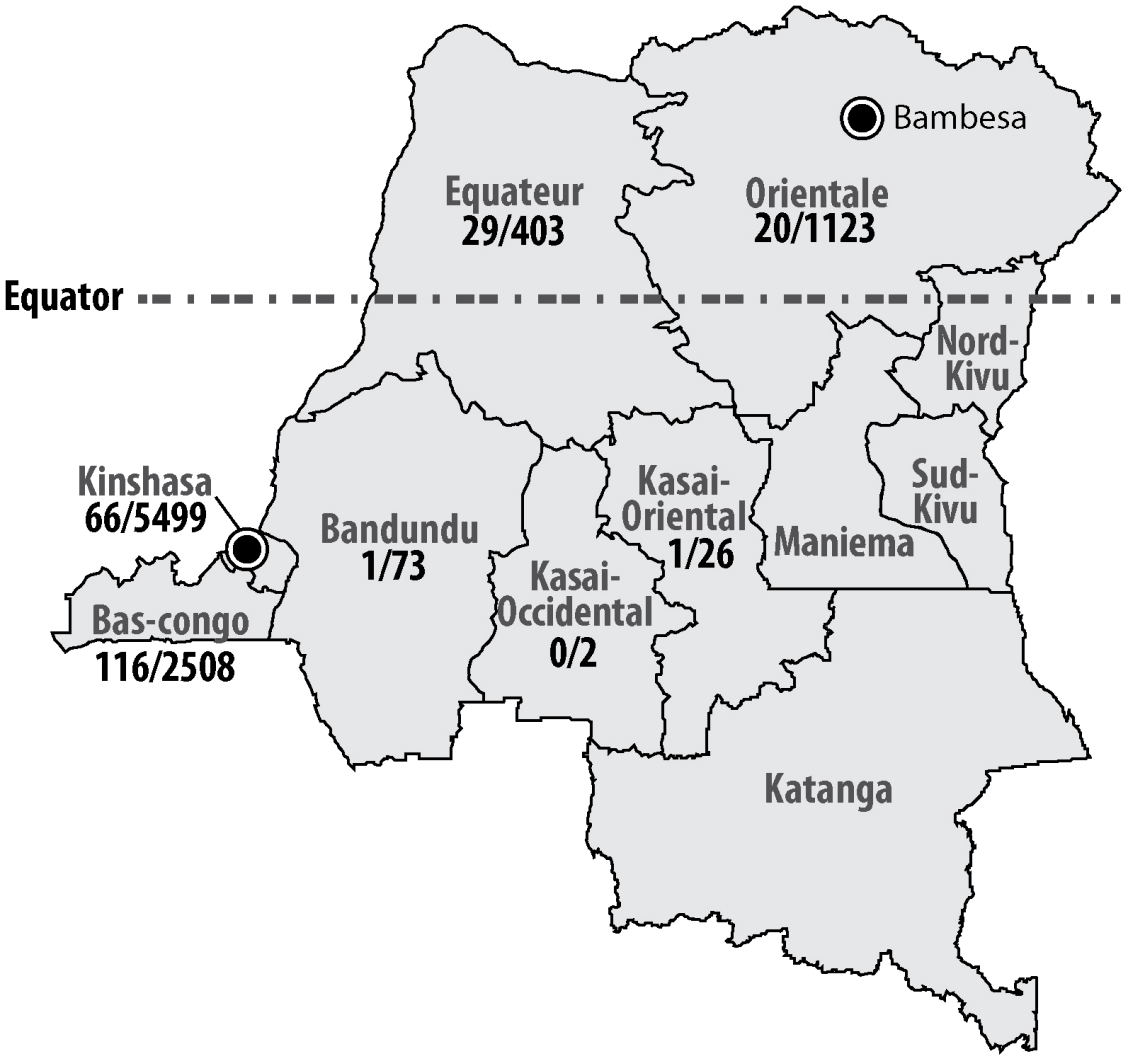
Origin of blood cultures and NTS in DR Congo (number of NTS grown/number of blood cultures received at INRB). Approximate positions of Kinshasa (4°23′S 15°26′E), Bambesa (3°27′N 25°43′E) and the equator are indicated.

**Figure 2:**
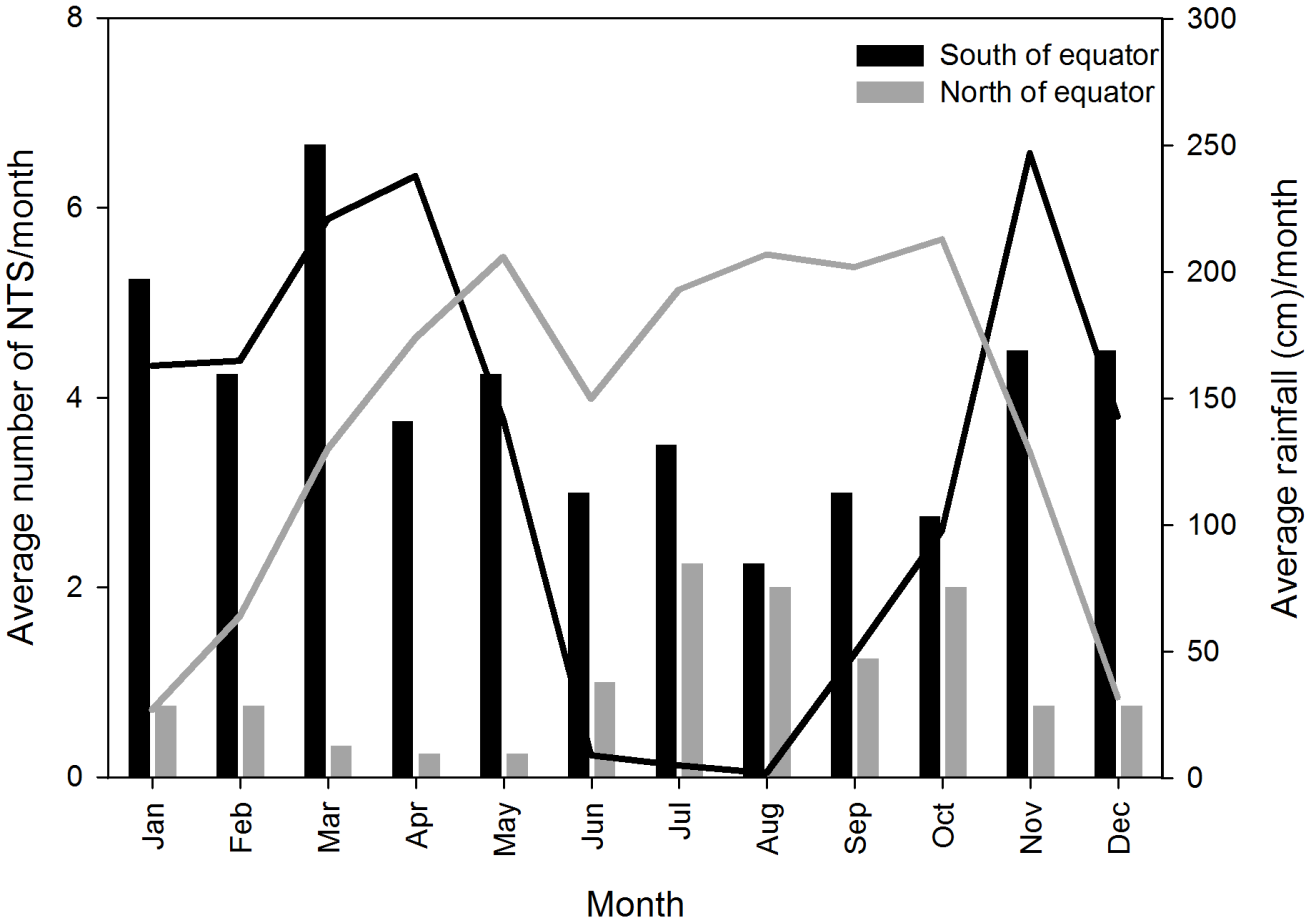
Average number of NTS per month south and north of the equator (black and grey bars respectively) and average monthly rainfall in Kinshasa (4°23′S 15°26′E, black line) and Bambesa (3°27′N 25°43′E, grey line).

The study was carried out in seven out of eleven provinces of DR Congo (Figure 1). In Kinshasa, health care facilities involved in the detection and study of the epidemic increase of typhoid fever-associated peritonitis of 2004 were selected [16]. Health care facilities in other provinces were recruited based on existence of microbiological laboratories, professional contacts and the accessibility to reliable shipment facilities. South of the equator (provinces of Bas-Congo, Kinshasa, Bandundu, Kasai Oriental, Kasai Occidental), the wet season runs from November till May, north of the equator (provinces of Equateur and Orientale), the wet season runs from April till October. Rainfall data were retrieved from http://www.dr-congo.climatemps.com/, using the data from Kinshasa (4°23′S 15°26′E) and Bambesa (3°27′N 25°43′E) as representative for the rainfall respectively south and north of the Equator (Figure 2). Malaria is endemic in DR Congo and 97% of the population is living in areas of stable transmission [17]. In adults aged 15–49 years old, the HIV prevalence rate in 2009 in DR Congo was estimated 1.2–1.6% [18]. The expanded vaccination program for children included the Bacille Calmette-Guérin at birth and live oral polio, diphtheria, whole cell pertussis, tetanus, hepatitis B, monovalent measles and *Haemophilus influenzae* b vaccines. Typhoid vaccine is not routinely administered [19].

### Bacterial culture and identification

Criteria for blood culture sampling were clinical suspicion of bacteremia caused by a local (pneumonia, urinary tract infection, meningitis or other) or systemic (typhoid fever, endocarditis) infection diagnosed at consultation or admission. Typhoid fever was defined according to the case definitions of the Ministry of Health surveillance of communicable diseases [20]. At the start of the surveillance project, teams of clinicians and laboratory technicians were trained in indications and sampling of blood cultures. Blood cultures of 9,634 patients presenting to health care facilities with clinical suspicion of bacteremia were sampled as part of a microbiological surveillance program between April 2007 and January 2011. For children <14 years, 1–4 ml of blood was sampled into pediatric blood culture vials (BacT/ALERT FP; bioMérieux; Marcy L'Etoile; France). For adults, 2×10 ml of blood was inoculated into aerobic blood culture vials (BacT/ALERT FA; bioMérieux; Marcy L'Etoile; France). Age, gender, geographic origin, use of antibiotics prior to sampling of the blood culture and presumptive diagnosis with suspected focus of bacteremia were recorded as part of the standard information present on the laboratory request forms. Vials were shipped at room temperature to the Institut National de Recherche Biomédicale (INRB) in Kinshasa (maximum delay of 6 weeks), where they were incubated at 35°C and daily checked for growth by visual inspection of the indicator. If grown, cultures were Gram stained, subcultured and identified to the species level by standard biochemical methods. Skin or environmental bacteria (coagulase negative staphylococci, *Corynebacterium* spp., *Propionibacterium acnes* and *Bacillus* spp.) were categorized as contaminants, the other bacteria were considered as clinically significant organisms (CSO) [21].

Suspected colonies of *Salmonella* were identified as NTS using standard biochemical methods (characteristic aspect on Kligler Iron Agar (acid from glucose, gas, production of H_2_S), negative tests for urease, oxidase, β-galactosidase and indole production tests, positive tests for lysine decarboxylase and the serotype was determined with commercial antisera (Remel, Lenexa, Kansas). Identity of *Salmonella* species isolates was confirmed using the Vitek II system (Card GN21 341, bioMérieux). At the National Reference Laboratory for *Salmonella* and *Shigella* (Institute of Public Health, Brussels), the serotype of the *Salmonella* isolates was re-confirmed by slide agglutination with commercial monospecific antisera (Sifin, Berlin, Germany), following the Kauffmann-White scheme [22].

For analysis in the present study, only the first isolate per patient was considered.

### Antimicrobial susceptibility

Susceptibility tests for ampicillin, cefotaxime and TMP-SMX and ESBL were performed using the Vitek II system (Card AB AST-N156, bioMérieux). For nalidixic acid, ciprofloxacin, chloramphenicol and azithromycin, minimal inhibitory concentration (MIC) values were determined using the E-test macromethod (bioMérieux). Breakpoints for resistance are summarized in table 1 and were according to Clinical Laboratory Standard Institute guidelines [23]. For ciprofloxacin and azithromycin, European Committee on Antimicrobial Susceptibility testing (EUCAST) V 2.0. guidelines [24] were followed. Strains co-resistant to ampicillin, chloramphenicol and TMP-SMX were considered as multidrug resistant (MDR) [25]. ESBLs flagged by the Vitek II system were phenotypically confirmed by the combined double-disk method using cefotaxime, ceftazidime and cefepime alone and in combination with clavulanic acid (Rosco Diagnostica, Taastrup, Denmark) according to CLSI guidelines and using *Escherichia coli* ATCC 25922 and *Klebsiella pneumoniae* ATCC 700603 as control strains [23]. Screening for mutations causing decreased ciprofloxacin susceptibility was performed by amplification and sequencing of the quinolone resistance-determining regions (QRDRs) of the *gyrA*, *gyrB*, and *parC* genes. The presence of the plasmid-mediated quinolone resistance *qnr* genes (*qnrA*, *qnrB*, and *qnrS*) was determined using PCR [26]. In confirmed ESBLs, the beta lactamase resistance genes were identified using a DNA microarray (Check-Points CT 101, Wageningen, the Netherlands) and PCR sequencing [27].

**Table 1:** Number blood cultures (BC) and number of *Salmonella* Typhimurium (T), *Salmonella* Enteritidis (E) and other NTS (O) by province and year of isolation.

Province		2007			2008			2009			2010+2011^a^			Total		
	BC	T	E	O	T	E	O	T	E	O	T	E	O	T	E	O
Bas Congo	2508	1	0	0	28	1	1	36	2	1	35	11^b^	0	100	14	2
Kinshasa	5499	13	8	2	24	5	1	5	1	0	5	2	0	47	16	3
Bandundu	73	0	0	0	1	0	0	0	0	0	0	0	0	1	0	0
Equateur	403	0	0	0	14	4	0	5	1	0	5	0	0	24	5	0
Kasai Occidental	2	0	0	0	0	0	0	0	0	0	0	0	0	0	0	0
Kasai Oriental	26	0	0	0	0	1	0	0	0	0	0	0	0	0	1	0
Orientale	1123	0	0	0	3	0	0	6	3	0	3	3	1	12	6	2
Total	9634	14	8	2	69	12	2	52	7	2	49	15	1	184	42	7

a January 2011 only.

b Onset of an epidemic outbreak in Kisantu, province of Bas-Congo [11].

### Pulsed field gel electrophoresis

Respectively 34 isolates of *Salmonella* Typhimurium and 16 of *Salmonella* Enteritidis were selected for pulsed field gel electrophoresis (PFGE). These isolates were representative according to geographic origin and year of isolation. PFGE was performed at the Institute of Public Health (IPH, Brussels) according to the Pulsenet protocol for *Salmonella* [28], using *XbaI* as restriction enzyme (New England Biolabs, Leusden, The Netherlands). Cluster analysis was performed with Bionumerics 5.1 (Applied Maths NV, Sint-Martens-Latem, Belgium), with as comparison settings the Dice similarity coefficient and UPMGA dendrogram type (optimization 0.50%, position tolerance 1.50%). The obtained PFGE profiles were compared to PFGE profiles of *Salmonella* Typhimurium or *Salmonella* Enteritidis originating from Belgium (n = 15 and n = 7 respectively) and Cambodia (n = 4 and n = 7 respectively), already stored in the IPH database.

### Data analysis

All data were entered in an Excel database (Microsoft Corporation, Redmond, Washington, USA). Proportions were assessed for statistical significance using the Chi square test or Fisher exact test, considering *p*<0.05 as significant. If data were normally distributed, mean values of two groups were compared with the t-test, otherwise median values were compared with the Mann-Whitney Rank Sum Test (Stata 10, StatCorp, Texas, USA).

## Results

### Serotypes, demographic, clinical and epidemiological data

From 9,634 blood cultures performed, 989 clinically significant organisms (CSO) were grown (10.3%). The proportion of CSO/blood cultures ranged between a minimum of 7.2% in the age group of 30–39 and a maximum of 11.9% in the age group of 0–9 years old [29]. With 233 isolates (23.6%), NTS ranked first among the CSOs, followed by *Salmonella* Typhi (20.3%) [29]. Non typhoid *Salmonella* consisted of *Salmonella* Typhimurium (n = 184, 79.0%), *Salmonella* Enteritidis (n = 42, 18.0%) and other *Salmonella* serotypes [n = 7, 3.0%: *Salmonella* Kisangani (n = 2), *Salmonella* Virchow (n = 2), *Salmonella 4,5* (n = 2) and a single isolate that could not be serotyped (Table 1)]. Most NTS (respectively 28.3 and 49.8%) originated from the provinces of Kinshasa and Bas Congo (Figure 1), in line with the higher number of blood cultures recovered from these provinces. Eleven out of a total of 42 *Salmonella* Enteritidis were isolated in Bas-Congo in 2010–2011, at the onset of an epidemic outbreak described in detail elsewhere [11].

Peaks in numbers of NTS fell in the rainy season (Figure 2). South of the equator, the rainy season from November till May, accounted for more NTS (mean of 4.7±1.0/month) than the dry season (mean of 2.9±0.5 NTS/month, *p* = 0.003). North of the equator, more NTS were observed from June till October (mean of 1.7±0.5 NTS/month), than in the rest of the year (mean of 0.57±0.3 NTS/month, *p*<0.001).

**Figure 3:**
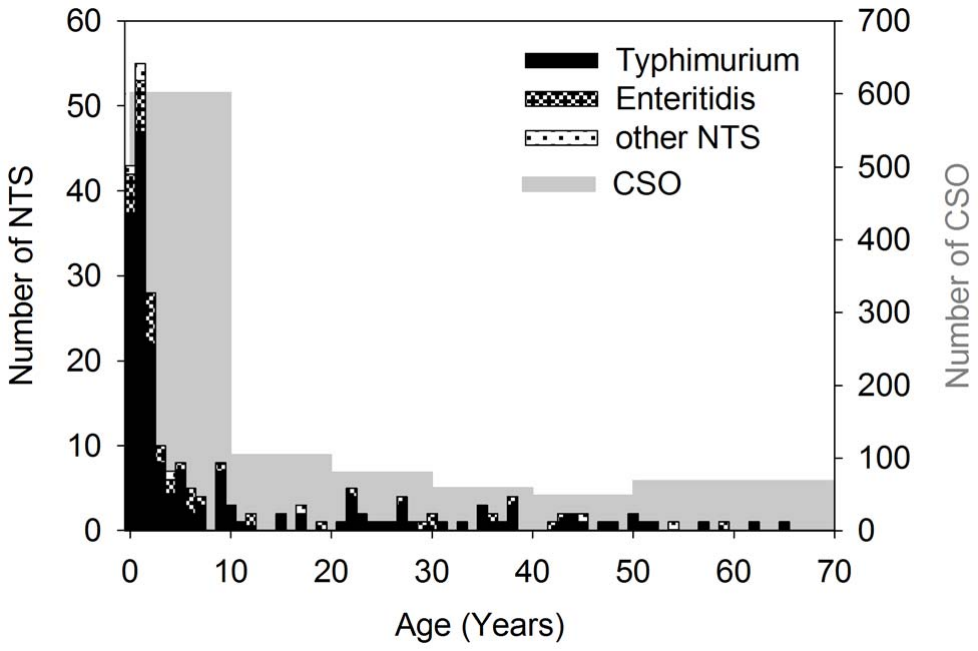
Age distribution of NTS (black bars) and number of clinical significant organisms. CSO<50: by age groups of 10 years, ≥50 was considered as one age group.

Patients' male/female ratio was 1.23 (125 male, 102 female, 6 no data). The age distribution of patients with NTS is illustrated in figure 3. The median patient age was 2 (interquartile range 1–11). There was no statistically significant difference between the median age of *Salmonella* Typhimurium and *Salmonella* Enteritidis patients (*p* = 0.1, Mann-Whitney Rank Sum Test). The presumptive diagnosis at the moment of sampling for the patients with NTS (in some patients more than one diagnosis was mentioned) was mainly typhoid fever (60.5%, 141/233), pneumonia (11.2%, 26/233), complicated urinary tract infection (7.7%, 18/233), meningitis (6.4%, 15/233), malaria (4.3%, 10/233), or non-specified other causes of bacteremia (14.6%, 34/233); for 8 patients (3.4%) no data were available.

A total of 40.8% (95/233) of NTS positive patients had taken antibiotics less than 48 hours prior to blood sampling.

### Antimicrobial susceptibility

The antimicrobial resistance profiles, MIC ranges, MIC50 and MIC90 for all NTS are shown in table 2, as well as the resistance profiles for *Salmonella* Typhimurium and *Salmonella* Enteritidis separately.

**Table 2:** Minimal inhibitory concentrations and antimicrobial resistance in 233 non-typhoidal *Salmonella* from DR Congo.

	Breakpoint MIC	NTS MIC range	NTS MIC50	NTS MIC90	% resistant NTS n = 233	% resistant *Salmonella* Typhimurium n = 184	% resistant *Salmonella* Enteritidis n = 42
Ampicillin	≥32	≤2–≥32	≥32	≥32	88.0%	94.0%	64.3%
Chloramphenicol	≥16^a^	2–>256	>256	>256	83.7%	90.2%	61.9%
TMP-SMX	≥4/76	≤1/19–≥16/304	≥16/304	≥16/304	88.0%	94.0%	64.3%
Nalidixic acid	≥32	2–>256	3	6	4.3%	4.9%	0.0%
Ciprofloxacin	>0.064^b^	0.006–0.19	0.012	0.023	4.3%	4.9%	0.0%
Azithromycin	>16^b^	2–256	4	12	3.0%	3.3%	0.0%
Cefotaxime	≥4	≤1–≥64	≤1	≤1	2.1%	2.2%	0.0%
ESBL	na	na	na	na	1.3%	1.1%	0.0%
MDR	na	na	na	na	80.7	87.0%	59.5%
MDR+DCS	na	na	na	na	3.9%	4.3%	0.0%
MDR+DCS+ESBL	na	na	na	na	0.9%	0.5%	0.0%

MIC: Minimal Inhibitory Concentration (mg/l); TMP-SMX: trimethoprim/sulphamethoxazole; MDR: multidrug resistant (Co-resistance against ampicillin, chloramphenicol and trimethoprim/sulphamethoxazole), DCS: decreased ciprofloxacin susceptibility; na not applicable.

a intermediate susceptible isolates were considered together with the resistant isolates;

b [24].

Respectively 88.0%, 83.7% and 88.0% (n = 205, 195 and 205) of all 233 NTS were resistant to ampicillin, chloramphenicol or TMP-SMX. The proportions of *Salmonella* Typhimurium resistant to ampicillin, chloramphenicol or TMP-SMX and MDR were significantly higher (*p*<0.001) compared to those among *Salmonella* Enteritidis. Decreased ciprofloxacin susceptibility was observed in 4.3% of isolates (*Salmonella* Typhimurium n = 9 and *Salmonella* 4,5 n = 1), all were nalidixic acid resistant and vice versa. In total 3.0% (7/233) of NTS were resistant to azithromycin: the isolates belonged to serotype *Salmonella* Typhimurium (n = 6) and *Salmonella* 4,5 (n = 1). Resistance to cefotaxime occurred in 2.1% of NTS (5/233, *Salmonella* Typhimurium n = 4 and *Salmonella* 4,5 n = 1). Vitek II labeled 9 strains as ESBL producers, only 3 (1.3%, *Salmonella* Typhimurium n = 2 and *Salmonella* 4,5 n = 1) were confirmed ESBL producers by double disk diffusion.

Multidrug resistance occurred in 80.7% of NTS (188/233). Combined MDR and decreased ciprofloxacin susceptibility was observed in nine strains. Two isolates (0.9%, one *Salmonella* Typhimurium and one *Salmonella* 4,5) combined MDR, decreased ciprofloxacin susceptibility, azithromycin and cefotaxime resistance and were ESBL producers. Only 10.3% of NTS isolates (8 *Salmonella* Typhimurium, 14 *Salmonella* Enteritidis, 1 *Salmonella* Kisangani and 1 *Salmonella* Virchow) were fully susceptible to all drugs tested.

In *Salmonella* Enteritidis isolated in Kinshasa (n = 16) significantly less MDR occurred (respectively 37.5% versus 73.1%, *p* = 0.02) than in *Salmonella* Enteritidis isolates recovered in the rest of DR Congo (n = 26). For *Salmonella* Typhimurium, this difference in MDR was not observed. However, *Salmonella* Typhimurium isolated from Kinshasa (n = 47) had a significantly higher proportion of azithromycin resistance (respectively 10.6% versus 0.7%, *p* = 0.005) than those isolated elsewhere (n = 137). For cefotaxime resistance in *Salmonella* Typhimurium, the difference between Kinshasa and the rest of DR Congo (respectively 6.4% versus 0.7%, *p* = 0.052) did not reach statistical significance. No difference in decreased ciprofloxacin susceptibility was seen in *Salmonella* Typhimurium between Kinshasa and other provinces (4.5 and 4.2%). All 3 ESBL producing isolates were recovered in Kinshasa.

Intake of antibiotics less than 48 hours prior to blood sampling was not associated with MDR, cefotaxime or azithromycin resistance (*p*>0.5), but was associated with decreased ciprofloxacin susceptibility (*p* = 0.03).

All 10 decreased ciprofloxacin susceptible isolates had mutations in the *gyrA* gene at position Asp87 that changed into Tyr (n = 8) or, in two isolates that were as well ESBLs, in Asn (n = 2). No mutations were observed in the *gyrB* or *parC* genes, nor were *qnrA*, *qnrB*, or *qnrS* genes detected in any of the 10 isolates. All 3 ESBL positives in double disk diffusion were identified by CheckPoints as SHV-2-like ESBLs and were confirmed as SHV-2a by PCR-sequencing.

### Pulsed field gel electrophoresis

Among the 34 isolates of *Salmonella* Typhimurium and 16 of *Salmonella* Enteritidis analyzed in PFGE, respectively 19 and 10 different PFGE profiles were observed (Figure S1). For *Salmonella* Typhimurium, PFGE profile T4 was most prevalent occurring in 20.6% (7/34) of isolates from DR Congo. This profile also occurred in 40% (6/15) of *Salmonella* Typhimurium isolates from patients in Belgium stored in the IPH database. Typhimurium profile T3 (2/34) was also observed in a *Salmonella* Typhimurium isolate from Belgium. For *Salmonella* Enteritidis, profile E5 was most common (25.0%, 4/16). Profile E2, observed in 2 isolates from DR Congo, corresponded to one of the common *Salmonella* Enteritidis profiles also observed in Belgium. The 11 NTS isolates from DR Congo with PFGE profiles identical to Belgian profiles originated from Bas-Congo (n = 5), Kinshasa (n = 4), Bandundu (n = 1) and Equateur (n = 1) and were isolated in 2008 (n = 8), 2010 (n = 2) and 2011 (n = 1).

## Discussion

The present study confirmed widespread MDR and low level decreased ciprofloxacin susceptibility and azithromycin resistance among NTS isolates from DR Congo, and demonstrated occurrence of ESBLs in *Salmonella* in Central Africa. The majority of isolated NTS consisted of *Salmonella* Typhimurium, it mainly affected infants and young children ≤2 years old and peaked in the wet season.

Some limitations are inherent to the study. The geographical origin of the isolates could have been biased by logistic difficulties such as limited road communications, especially during the rainy season. Although we cannot completely exclude that shipment delays of several days to weeks might have resulted in lower recovery rates, preliminary survival studies suggested that Gram negative bacteria remain viable for at least 8 weeks in inoculated and grown blood culture bottles in the environmental conditions encountered in DR Congo. Given antibiotic use prior to sampling, the inherent low sensitivity of blood culture [30], financial constraints preventing many patients from consulting, unfamiliarity of medical doctors with microbiological culture tools and possible variability in sampling criteria between different sites in function of time (*e.g.* incomplete sampling during evening and night shifts) and variability in training of clinicians, and in function of other diagnosis made (*e.g.* tendency not to take blood culture when thick blood film is *Plasmodium* positive in children is likely to under-represent NTS further), we believe that the presently described isolates represented only a fraction of the actual number of *Salmonella* infections and that calculations of NTS incidence rates is not possible based on our data. Although only an association for decreased ciprofloxacin susceptibility was observed with antibiotic intake (which consisted mainly of first line drugs), we cannot exclude some bias towards resistance. Besides *qnrA*, *qnrB*, and *qnrS*, the presence of other plasmid-mediated quinolone resistance genes was not determined nor was the *parE* gene sequenced, taking into account the low probability of mutations in the *parE* gene in *parC* and *gyrB* wild type *Salmonella*. Other mechanisms associated with decreased ciprofloxacin susceptibility can therefore not be completely excluded. Furthermore, we limited the number of PFGE analysis to a subset of representative strains in function of the moment and region of isolation, taking into account the relatively homogeneous PFGE profile observed earlier with *Salmonella* Typhi and *Salmonella* Typhimurium strains isolated in DR Congo [8], [29].

We were able to cover antimicrobial resistance patterns in a large surface of DR Congo (7 out of 11 provinces), where the NTS situation was hitherto poorly characterized. Indeed, previous data about NTS bacteremia in DR Congo comprise 6 studies only, performed over 35 years in 3 sites [6]–[9], [31], [32]. Comparison of these studies, although performed at sites almost 1000 km apart, illustrates the emergence of antimicrobial resistance in DR Congo. In the seventies, respectively 38 and 46% of *Salmonella* Typhimurium and *Salmonella* Enteritidis were reported as resistant [31]. In the eighties, antibiotic resistance was still only reported in 30% of NTS, but all tested isolates were sensitive to TMP-SMX [6]. In the early nineties, 100% resistance to ampicillin and chloramphenicol was already reported, and resistance to TMP-SMX started to emerge (7%), in the absence of resistance to fluoroquinolones or third generation cephalosporins [7]. The same report also mentions that in 1998–1999 resistance against TMP-SMX had increased to 90%, still in the absence of fluoroquinolone resistance. Also in the late nineties, over 50% of MDR was reported in Kinshasa [9]. Finally, in 2000–2006 in eastern DR Congo, 85–96% resistance to ampicillin, chloramphenicol, TMP-SMX and MDR, was observed and NTS resistance to fluoroquinolones was reported for the first time to be 3.8–6.1% [8]. These percentages, as predicted by the authors, are in line with those reported here for the whole country in 2007–2011, with the exception of resistance against third generation cephalosporin, which we observed for the first time. The antibiotic resistance profile we observed in DR Congo is very similar to the ones described in Togo [13], in Ghana, Kenya, Malawi and Mozambique [5], [33]–[35], although no 3^rd^ generation cephalosporin resistance was observed in the later four studies. In Nigeria on the other hand, resistance in *Salmonella* Enteritidis appears to be more elevated [36]. We observed a difference in resistance between *Salmonella* Typhimurium and *Salmonella* Enteritidis to first generation antibiotics, confirming data from Malawi [34]. As suggested by these authors, acquisition of MDR and increased prevalence may suggest competition for an ecological transmission niche between these two serotypes. Decreased use of first line drugs after a MDR *Salmonella* Typhi outbreak in Kinshasa [16] may have caused a loss of MDR selection pressure, contrary to other parts of DR Congo where ampicillin, chloramphenicol and TMP-SMX are still frequently used [8]. Increased use of cefotaxime and particularly azithromycin as alternative treatments in Kinshasa may have selected for the observed higher resistance of *Salmonella* Typhimurium against these drugs. Furthermore, in the present study, decreased ciprofloxacin susceptibility was in all cases associated with nalidixic acid resistance and a mutation at codon 87 of the *gyrA* gene. Detection of nalidixic acid resistance therefore still remains a valuable test to screen for decreased ciprofloxacin susceptibility in DR Congo, contrary to the Asian situation [37].

The SHV-2 type ESBL, which was identified in all 3 ESBL producing isolates has been observed in a minority of South African ESBL positive *Salmonella* spp (5%) [14], and SHV-2-like ESBLs have been identified in *Enterobacteriaceae* isolated from drinking water in Kinshasa previously [38].

The observed seasonal peak of NTS isolation, coinciding with the rainy season, has been described before [6] and may be explained by contamination of the surface waters.

Host risk factors for NTS such as increased incidences of malaria and malnutrition [3] that peak at the same moment, have already been described as relatively common in NTS patients in DR Congo [8]. Prevalence of HIV, another host risk factor for NTS, is low in DR Congo [18], which is as well reflected by the age distribution of NTS in children ≤2 years, similar as 25 years ago [6] and its low occurrence in adults.

Appearance in DR Congo of PFGE profiles which are common to Belgian profiles, might be explained, by exchange of food products [3]. For example, it is well documented that *Salmonella* is a pathogen occurring particularly in poultry products, and that human *Salmonella* infections may be attributable to the consumption of contaminated chicken [39]. DR Congo represents the twelfth export destination of European Union poultry meat products, representing 2.8% of the total European poultry meat exports [40], [41]. Travelling of asymptomatic carriers [42] between the two countries, circulation of major worldwide clones [43], or the limited technical power of PFGE to discriminate the clonal relation of organisms might be alternative explanations for appearance of identical PFGE profiles in Belgium and DR Congo [34], [44]. The obtained PFGE profiles and representative strains are available for comparisons with profiles obtained with *Salmonella* from other countries.

As we did not determine the genotype of *Salmonella* Typhimurium isolated from DR Congo, we do not know if it belongs to multilocus sequence type (ST) 313, which is geographically confined to sub-Saharan Africa and represents the predominant ST-type among invasive *Salmonella* Typhimurium strains in Malawi and Kenya [45]. ST313 genetically differs from other STs within the serotype by a degraded genome capacity – a feature which is also noted among *Salmonella* Typhi and *Salmonella* Paratyphi and which suggest adaptation of ST313 to the human host [45], [46]. As one of the two ST313 lineages circulating in East-Africa is hypothesized to have originated in DR Congo [46], it would be of interest to determine if the ST313 lineages was present, or absent, in the *Salmonella* Typhimurium strain collection.

Due to the high prevalence of MDR, especially in *Salmonella* Typhimurium which appears the most prevalent NTS in DR Congo, the use of cheap first line drugs seems not indicated since they will no longer be active. Third generation cephalosporins and azithromycin may still be effective although resistance is emerging. Close surveillance of NTS and their microbial resistance patterns seems indicated to follow-up on emerging resistance, for which the actual surveillance study already provides a baseline, to prevent outbreaks, and to further rationalize therapy.

Furthermore, research into the possible NTS transmission pathways [1] and incidence rates remains indicated to take preventive measures and see their effect.

## Supporting Information

Figure S1 **Pulsed-field gel electrophoresis (PFGE)** ***Xba*** **I patterns of 34** ***S.*** **Typhimurium (a) and 16** ***S.*** **Enteritidis (b) isolates from DR Congo.** Similarity between PFGE patterns was assessed by cluster analysis (Dice coefficient and UPGMA, tolerance and optimization of band position set at 1.5% and 0.5%). * PFGE profile also observed in *Salmonella* Typhimurium or *Salmonella* Enteritidis from Belgium.
(TIF)
