# Supplementary figures and images for: Antimicrobial Resistance in Invasive Non-typhoid Salmonella from the Democratic Republic of the Congo: Emergence of Decreased Fluoroquinolone Susceptibility and Extended-spectrum Beta Lactamases
Source: PLoS Negl Trop Dis. 2013 Mar 14;7(3):e2103. doi: 10.1371/journal.pntd.0002103 (PMC3597487; doi:10.1371/journal.pntd.0002103)

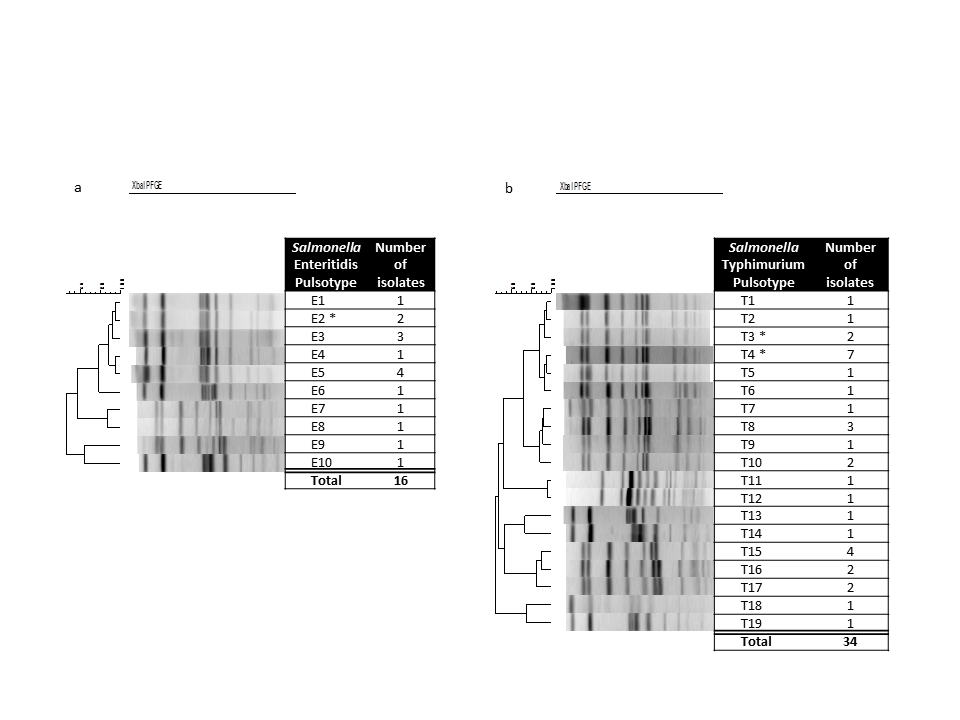

Supplement: Figure S1 — Pulsed-field gel electrophoresis (PFGE) Xba I patterns of 34 S. Typhimurium (a) and 16 S. Enteritidis (b) isolates from DR Congo. Similarity between PFGE patterns was assessed by cluster analysis (Dice coefficient and UPGMA, tolerance and optimization of band position set at 1.5% and 0.5%). * PFGE profile also observed in Salmonella Typhimurium or Salmonella Enteritidis from Belgium. (TIF) [file pntd.0002103.s001.tif]
